# Supplementary material for: A systematic review of the prediction of hospital length of stay: Towards a unified framework
Source: PLOS Digit Health. 2022 Apr 14;1(4):e0000017. doi: 10.1371/journal.pdig.0000017 (PMC9931263; doi:10.1371/journal.pdig.0000017)
Supplement: Attachment — Submitted filename: PLOS DIGITAL HEALTH REBUTTAL LETTER.docx [file pdig.0000017.s003.docx]

# PLOS Rebuttal Letter: Response to Reviewers

Martin G Frasch

Section Editor

PLOS Digital Health

Submission Date: 15/11/21

Dear Professor Frasch,

Thank you for inviting us to submit our revised manuscript entitled, "The Prediction of Hospital Length of Stay: Towards a Unified Framework" to PLOS Digital Health. We also appreciate the time and effort you and each of the reviewers have dedicated to providing insightful feedback on ways to strengthen our paper. Therefore, it is with great pleasure that we resubmit our article for further consideration. We have incorporated changes that reflect the detailed suggestions provided. We also hope that our edits and the responses we provide below satisfactorily address all the issues and concerns you and the reviewers have noted.

To facilitate your review of our revisions, the following is a point-by-point response to the questions and comments delivered in your letter.

**EDITOR SUGGESTIONS:**

1. *“We note that your submission is a Systematic or Scoping Review, which should be uploaded as a 'Research Article' article type in Editorial Manager. Please change the article type to 'Research Article’ and ensure all submission questions are completed for this article type. Please note that, if accepted, Research Articles have an APC associated with publication.”*
   - **RESPONSE: We have now uploaded this article as a “Research article”.**
2. *“Please ensure that the CRediT author contributions listed for every co-author are completed accurately and in full.
   At this stage, the following Authors require contributions: Kieran Stone, Reyer Zwiggelaar, Phil Jones, and Neil Mac Parthalain.  Please ensure that the full contributions of each author are acknowledged in the "Add/Edit/Remove Authors" section of our submission form.”*
   - **RESPONSE: We have now credited author contributions as suggested.**
3. *We ask that a manuscript source file is provided at Revision. Please upload your manuscript file as a .doc, .docx, .rtf or .tex. If you are providing a .tex file, please upload it under the item type ‘LaTeX Source File’ and leave your .pdf version as the item type 'Manuscript'.*
   - **RESPONSE: We have now added a LateX source file to the submission.**
4. *Please provide separate figure files in .tif or .eps format only, and remove any figures embedded in your manuscript file. If you are using LaTeX, you do not need to remove embedded figures.*
   - **RESPONSE: We have now provided separate figures in the appropriate format.**
5. *Please provide a complete Data Availability Statement in the submission form, ensuring you include all necessary access information or a reason for why you are unable to make your data freely accessible. Note that it is not acceptable for the authors to be the sole named individuals responsible for ensuring data access.*
   - **RESPONSE: We have now completed a Data Availability statement as part of the submission.**
6. *Please amend your detailed Financial Disclosure statement. This is published with the article, therefore should be completed in full sentences and contain the exact wording you wish to be published.*
   (i) *State what role the funders took in the study. If the funders had no role in your study, please state: "The funders had no role in study design, data collection and analysis, decision to publish, or preparation of the manuscript."*
   (ii) *If any authors received a salary from any of your funders, please state which authors and which funders.*
   (iii) *If you did not receive any funding for this study, please simply state: "The authors received no specific funding for this work."*

**RESPONSE: We have now amended the Financial disclosure statement.**

**Important note for reviewers:

In the marked-up copy of the manuscript, colours are coded to each reviewer. Red is coded to Reviewer 1, Blue to Reviewer 2 and Purple to Reviewer 3.**

**REVIEWER 1 COMMENTS:**

1. *“The abstract and introduction present this paper as a systematic review (or rapid review). However, the structure and content of the paper do not match a systematic review usual structure, despite the mention of the PRISMA checklist. For example, the methods are briefly described as a sub-section of the introduction (not in a separate section), and there is no ‘findings’ section as such, separate from discussion.”*

**RESPONSE: Thank you for this comment, we have now corrected these sections to be more in line with a typical systematic review and the methods are no longer a subsection of the introduction. We have now included a brief findings section which points to the main findings of the paper which are presented as a discussion in Section 6 where we encapsulate the relative shortcomings of LoS prediction research. The studies that were chosen for this paper were put through a rigorous selection process that is now presented on pages 3-6.**

2. *“The methods subsection misses some essential information about the review process. First of all, it mentions the search for (and inclusion of) ‘surveys’ – from which papers are retrieved, and then studies included. This is very confusing. It is not known what ‘surveys’ mean in this context. Are they systematic reviews? Is this a systematic review of systematic reviews? It also does not describe the review process: e.g. inclusion/exclusion criteria; the search strategy; criteria for screening results; methods for data extraction; how many researchers involved in the screening (reliability); methods for analysis… It is impossible to assess the quality of this study without this information.”*

**RESPONSE: Thank you for your comment. The term ‘surveys’ represents a review of a large number of papers within the field in question, these are not exclusively limited to systematic reviews. We describe having found and reviewed 32 survey papers using the search terms described in the paper. We extract 220 papers that were identified to be relevant to hospital length of stay prediction. This is not a systematic review of reviews it merely uses other reviews/surveys and their reference lists as a source for examination within this manuscript. Furthermore, we appreciate your guidance when it comes to the manuscript structure and we have included the necessary information in alignment with your comment, so that the quality of this study can be better assessed. This has been clarified in more detail in pages 3-6**

3. *“The abstract and introduction also suggest that the analysis is done with a unifying framework. However this is not the case – (the need for) a framework is only presented, it seems, as a conclusion.”*

**RESPONSE: Thank you for your comment. The analysis in this systematic review is not performed using a unified framework. The purpose of this paper is to review literature surrounding hospital length of stay prediction and highlight with discussion that there is no common ground or framework utilized to predict length of stay. Instead, each modelling approach is only employable within the environment it was made and is usually disease, condition or patient-group specific. As a result of this, we propose a unified framework in order to address this issue within the field. Using such a framework would enable comparison of performance for length of stay models in the literature as well as making models more generalizable in a variety of hospital settings. A unified framework is not used for this systematic review because it doesn’t directly consider the prediction of length of stay data and instead reviews approaches that predict length of stay. Thus, as you have said, the need for a framework is presented as a conclusion. We have reflected these aspects in pages 26-30.**

4. *“Overall this paper feels as materials written for, or derived from, another document, such as a thesis, book chapter or project report. It also makes reference to an Appendix which is not provided in this submission (I could not find it).
   I suggest the authors rewrite this paper in the structure of a systematic review. The topic is potentially interesting and worth publishing*.”

**RESPONSE: Thank you for this kind comment. We have chosen to rewrite the paper in the structure of a systematic review as you suggested. The appendix has now been labelled appropriately and can be found at the very end of the paper. The appendix is visible on the final page of the paper.**

**REVIEWER 2 COMMENTS:**

1. *“Since this manuscript is a review paper, it would be nice to have a table organized as a whole so that it is easy to find each reference. In other words, a classification table such as category for the final extracted 93 studies is required (similar to Table 5). For example, I recommend adding a table matching the reference with the taxonomy created in Figure 2”*

**RESPONSE: Thank you for this suggestion. We have now added a table for ease of navigation on page 6 of the manuscript.**

2. “*The order of the subsection of Chapter 2 is 2.1 Operational research based approaches, 2.2 Statistical and Arithmetic approaches, 2.3 Machine Learning and Data Mining approaches. Therefore, the taxonomy in Figure 2 should also be arranged in the order of subsections. Currently, the name and order are different.”*

**RESPONSE: Thank you for this suggestion, we have now corrected the taxonomy appropriately.**

3. *“Sections 2.3, 2.3.1, and 2.3.2 do not match the name and structure of Figure 2. Therefore, either modify the machine learning part of the taxonomy or modify this section according to the structure of the taxonomy.”*

**RESPONSE: Thank you for the suggestion, we have now corrected this so that there is no conflict and the machine learning part of the taxonomy has been modified.**

4. *“Chapter 4 has only one section in section 4.1, so either add content to make it after section 4.2 or remove the title of section 4.1 and combine it into section 4.”*

**RESPONSE: Thank you for the suggestion, we have now combined the subsection into Section 5 removing 5.1 (previously Section 4 and 4.1) as reflected on page 25**

5. *“In this manuscript, the papers up to 2019 were well organized, but it seems that studies using more diverse techniques for machine learning were conducted in 2020 and 2021. I think that, at least, machine learning techniques using the latest deep learning need to be added.”*

**RESPONSE: Thank you for the suggestion, we have now included 5 additional studies that were conducted in 2020 and 2021. These are covered on pages 16,17,19 and 20.**

**REVIEWER 3 COMMENTS:**

1. *“The ethical implications of modeling LOS are not adequately covered (i.e., in use could a health system use this type of modeling strategy to deny care to those perceived to be high utilizers?)”*

**RESPONSE: Thank you for this suggestion, we have now included some ethical implications of modeling length of stay on page 18.**

2. *“The inherent opportunities for bias using EHR data in terms of modeling approaches. We know that clinicians have inherent biases so tests ordered, assessments documented, will also carry the inherent bias of these processes and will be included in these models.”*

**RESPONSE: Thank you for this suggestion, we have now included a consideration of the bias of using EHR data on page 18.**

**CONCLUDING REMARKS**: Again, thank you for giving us the opportunity to strengthen our manuscript with your valuable comments and queries. We have worked hard to incorporate your feedback and hope that these revisions persuade you to accept our submission.

Sincerely,

Kieran Stone

Aberystwyth University

kis12@aber.ac.uk
